# Combining cohort and period methods for retrospective time trend analyses of long-term cancer patient survival rates

**DOI:** 10.1038/sj.bjc.6601295

**Published:** 2003-09-30

**Authors:** H Brenner, C Spix

**Affiliations:** 1 Department of Epidemiology, German Centre for Research on Ageing, Bergheimer Str. 20, D-69115 Heidelberg, Germany; 2 German Childhood Cancer Registry, Obere Zahlbacher Str. 69, D-55131 Mainz, Germany

**Keywords:** cohort analysis, period analysis, neoplasms, prognosis, registries, survival

## Abstract

Assessing trends in long-term cancer patient survival is an essential component of monitoring progress against cancer by cancer registries. Traditional assessment of long-term survival (‘cohort analysis’) is very useful to disclose trends in long-term survival rates of patients diagnosed many years ago, but it does not allow the disclosure of recent trends in long-term survival rates. The latter can be achieved by an alternative method of survival analysis (‘period analysis’), which has been proposed a few years ago. On the other hand, unlike cohort analysis, period analysis does not provide estimates of long-term survival rates for patients diagnosed in the early years after initiation of cancer registration. In this paper, a method of retrospective analysis of time trends in long-term survival rates is introduced, which combines the advantages of both cohort and period analysis (‘mixed analysis’). This method thereby allows for a comprehensive monitoring of trends in long-term survival over an extended time span from the earliest to the most recent years of cancer registration. The use of the method is illustrated for retrospective time trend analyses of long-term survival of cancer patients in the United States with the 1973–1999 database of the Surveillance, Epidemiology, and End Results Program of the National Cancer Institute.

Monitoring trends in long-term cancer patient survival rates is an essential component of cancer surveillance. Long-term cancer survival statistics are now reported by an increasing number of cancer registries around the world (e.g., Sankaranarayanan *et al*, 1998; Berrino *et al*, 1999). In the past, long-term survival rates have mostly been calculated in a ‘cohortwise’ manner, that is, for cohorts of patients diagnosed in certain calendar years for whom long-term follow-up, such as 5-, 10-, or 20-year follow-up, has been completed in the meantime. Cohortwise analyses are very useful for retrospective analyses of survival trends among those cohorts of patients, but they cannot be applied to more recently diagnosed patients for whom long-term follow-up is not yet available.

To provide more recent estimates of long-term survival, an alternative method, denoted ‘period analysis’, has been proposed a couple of years ago (Brenner and Gefeller, 1996, 1997). With this method, recent survival estimates can be obtained by restricting the analysis to the survival experience of patients within some recent time interval (which is achieved by left truncation of observations at the beginning of that interval in addition to right censoring at its end). Period estimates derived in that way quite closely predict long-term survival rates observed many years later for patients diagnosed in the period of interest, thereby enabling early detection of recent trends (Brenner and Hakulinen, 2002a,2002b; Brenner *et al*, 2002b). On the other hand, period estimates of long-term survival cannot be derived for the first years after initiation of cancer registration, as their derivation requires that the database includes patients who have been under long-term observation in the period of interest.

In this paper, a method of retrospective analysis of time trends in long-term survival rates is introduced, which combines the advantages of cohort and period analysis (‘mixed analysis’). This method thereby allows for a comprehensive monitoring of trends in long-term survival over an extended time span from the earliest to the most recent years of cancer registration.

## METHODS AND RESULTS

### Time trend analysis of long-term cancer survival rates with the 1973–1999 SEER database

The method is illustrated for retrospective analyses of trends in long-term survival of cancer patients in the United States with the 1973–1999 database of the Surveillance, Epidemiology, and End Results Program of the National Cancer Institute (SEER, 2002). The SEER Program is the most authoritative source of information on cancer incidence and survival in the United States, and it is considered as the standard for quality among cancer registries around the world. Data included in the 1973–1999 SEER database are from nine population-based cancer registries, which together cover a population of about 24 million people (SEER, 2002).

The different approaches to retrospective time trend analyses of 10-year survival rates as applied to the 1973–1999 SEER database are illustrated in Table 1

**Table 1:** Analysis of time trends in 10-year survival rates in 1973–1999 by cohort, period, and mixed analysis

	**Cohort analysis**		**Period analysis**		**Mixed analysis**	
**Calendar year**	**Year of diagnosis**	**Survival experience**	**Year of diagnosis**	**Survival experience**	**Cohort analysis^a^**	**Period analysis^a^**
1973	1973	1973–1983			1–10	
1974	1974	1974–1984			1–10	
1975	1975	1975–1985			1–10	
1976	1976	1976–1986			1–10	
1977	1977	1977–1987			1–10	
1978	1978	1978–1988			1–10	
1979	1979	1979–1989			1–10	
1980	1980	1980–1990			1–10	
1981	1981	1981–1991			1–10	
1982	1982	1982–1992			1–10	
1983	1983	1983–1993	1973–1983	1983	1–10	
1984	1984	1984–1994	1974–1984	1984	1–10	
1985	1985	1985–1995	1975–1985	1985	1–10	
1986	1986	1986–1996	1976–1986	1986	1–10	
1987	1987	1987–1997	1977–1987	1987	1–10	
1988	1988	1988–1998	1978–1988	1988	1–10	
1989	1989	1989–1999	1979–1989	1989	1–10	
1990			1980–1990	1990	1–9	10
1991			1981–1991	1991	1–8	9–10
1992			1982–1992	1992	1–7	8–10
1993			1983–1993	1993	1–6	7–10
1994			1984–1994	1994	1–5	6–10
1995			1985–1995	1995	1–4	5–10
1996			1986–1996	1996	1–3	4–10
1997			1987–1997	1997	1–2	3–10
1998			1988–1998	1998	1	2–10
1999			1989–1999	1999		1–10

a Each column contains the range of years after diagnosis for which survival estimates were contributed by either cohort or period analysis; the cohort analysis part is derived from the cohort of patients diagnosed in the calendar year given in the leftmost column, whereas the period analysis part refers to survival experience in the calendar year 1999 in all cases.

. Whereas traditional cohort analysis allows the assessment of trends in 10-year survival rates for patients diagnosed between 1973 and 1989 only, the applicability of period analysis is restricted to the years from 1983 to 1999. For those years, period analysis provides the most up-to-date estimates of 10-year survival that would have been available within each year. In retrospective trend analyses, however, the empirically demonstrated use of period estimates for a given calendar year as a surrogate for the survival rates later observed for patients diagnosed in that year (Brenner and Hakulinen, 2002b; Brenner *et al*, 2002b) may only be meaningful for those calendar years for which cohort estimates are not available. In our example, this would pertain to the calendar years from 1990 to 1999. Furthermore, some part of the survival function may be estimated by cohort analysis even for patients diagnosed in those calendar years. In fact, except for the year 1999, period analysis would only be needed for completing the survival function over the full 10 years of interest. Hence, a combination of cohort analysis and period analysis in a ‘mixed analysis’ may be useful for retrospective trend analysis of long-term survival (see the two columns on the right-hand side of Table 1).

For example, an estimate of 10-year survival for patients diagnosed in 1990 may be obtained by combining their survival experience during the first 9 years following diagnosis, which can be obtained by cohort analysis, with the most recent (1999) period estimate of conditional survival for the 10th year following diagnosis. That is, survival in the 10th year would be estimated using survival experience in the 10th year of follow-up in 1999 of patients diagnosed in 1989 and 1990. Similarly, 10-year survival for patients diagnosed in 1991 may be estimated by combining their survival experience during the first 8 years following diagnosis, which is obtained by cohort analysis, with the most recent (1999) period estimates of conditional survival for the 9th and the 10th year following diagnosis. For more recent cohorts, an increasing part of the survival function has to be obtained by period analysis, and the 10-year survival estimate for the 1999 cohort is exclusively obtained by period analysis. Thus, while a retrospective time trend analysis by cohort and period analysis would be restricted to the 17-year time intervals 1973–1989 and 1983–1999, respectively, ‘mixed analysis’ allows a comprehensive time trend analysis over the full 27-year time span from 1973 to 1999.

Obviously, the value of ‘mixed analysis’ increases with increasing length of the follow-up of patients (see Table 2

**Table 2:** Calendar years for which 5-, 10-, 15-, and 20-year survival rates can be obtained by cohort, period, and mixed analysis using the 1973–1999 SEER database

	**Cohort analysis**	**Period analysis**	**Mixed analysis**
5-year survival	1973–1994	1978–1999	1973–1999
10-year survival	1973–1989	1983–1999	1973–1999
15-year survival	1973–1984	1988–1999	1973–1999
20-year survival	1973–1979	1993–1999	1973–1999

). For example, analyses of 15-year survival rates by cohort and period analysis would provide time trends for nonoverlapping 12-year time intervals from 1973 to 1984 and from 1988 to 1999 only, respectively. Analyses of trends in 20-year survival rates would even be restricted to 7-year time intervals with cohort analysis (1973–1979) and period analysis (1993–1999), whereas a trend analysis of long-term survival over the full 27 years can only be obtained by mixed analysis for all types of survival estimates.

### Empirical examples

The different types of analyses are illustrated for time trends in 5-, 10-, 15-, and 20-year survival rates of patients with one of three very common forms of cancer (colon cancer, lung cancer, breast cancer) in the United States using the 1973–1999 SEER database. In addition, analyses are shown for patients with testicular cancer, as this cancer typically occurs at a relatively young age, in which case long-term survival rates are of particularly high interest. Data are presented for all races, all ages, and (for colon and lung cancer) both sexes combined. Patients with a prior diagnosis of cancer were excluded, as were patients whose cancer was known by death certificate only (less than 2% for all types of cancer included in this analysis) or by autopsy only (less than 1% for all types of cancer included in this analysis).

All presented survival figures are relative rather than absolute survival rates. The relative survival rates reflect ‘net survival’ of patients with cancer. They can be interpreted as the expected survival experience of cancer patients in the hypothetical situation in which the particular cancer is the only cause of death (Ederer *et al*, 1961). The relative survival rates are calculated as the ratio of absolute survival rates of cancer patients divided by the expected survival rates of a group of patients of the corresponding age and sex in the general population. Estimates of expected survival rates were derived from pertinent population life tables according to Hakulinen's (1982) method.

**Figure 1:**
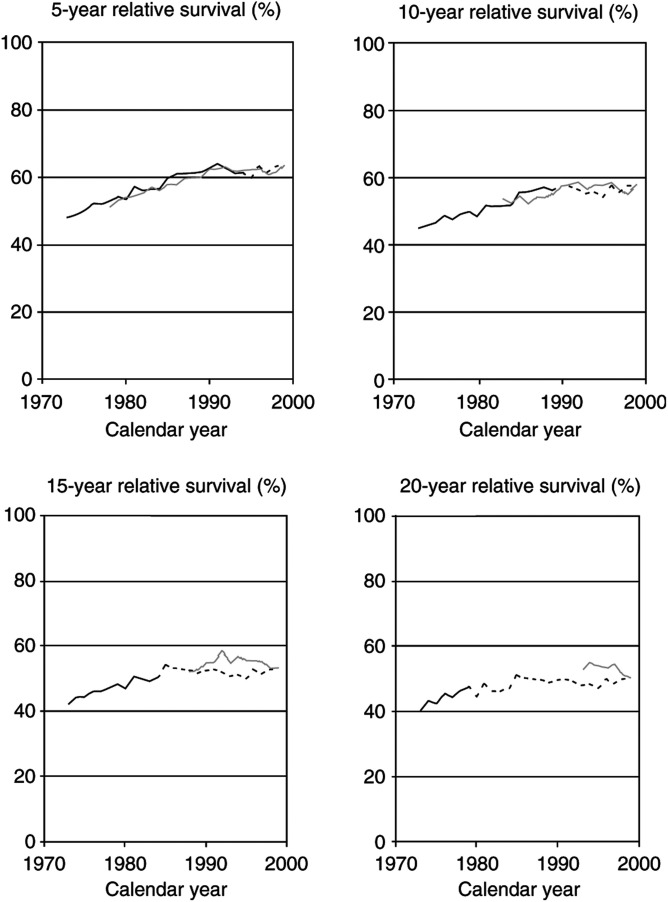
Trends in 5-, 10-, 15-, and 20-year relative survival rates of patients with a first diagnosis of colon cancer in the United States according to cohort analysis (solid black lines), period analysis (solid grey lines), and mixed analysis (dotted extension of black lines). SEER 1973–1999 database, all races, both sexes combined.

Trends in 5-, 10-, 15-, and 20-year survival rates of colon cancer patients as estimated by cohort analysis (solid black lines) and period analysis (solid grey lines) are shown in Figure 1

. Both cohort and period analyses indicate an upward trend in long-term survival rates in 1973–1999, but evidence from cohort analysis is restricted to patients diagnosed in the earlier years, whereas evidence from period analysis is restricted to the later years. Only for the 5- and 10-year survival trends, there is some overlap of the time frame encompassed by both approaches. Within those overlapping time intervals, estimates from cohort and period analysis are in general quite close, with a tendency towards slightly lower estimates from period analysis as expected from both theory and previous extensive empirical evaluation (Brenner and Hakulinen, 2002b; Brenner *et al*, 2002b). This means that an analysis of long-term survival conducted by period analysis in those years would have underestimated survival as estimable by now only slightly.

A more comprehensive picture of time trends in long-term survival rates of patients with colon cancer is provided by mixed analysis, which combines elements of both cohort and period analysis as outlined in Tables 1 and 2. With this approach, trend curves are available over the entire 1973–1999 time span, as indicated by the dotted extensions of the black trend lines up to and including the year 1999 (in which mixed analysis is equal to period analysis by definition). This analysis demonstrates that long-term relative survival rates of patients with colon cancer have substantially increased over time until the middle of the 1980s, whereas long-term relative survival estimates have remained rather constant (at levels slightly above 60% for 5-year survival, and between 50 and 60% for 10-, 15-, and 20-year survival) for patients diagnosed in later years.

Figures 2

**Figure 2:**
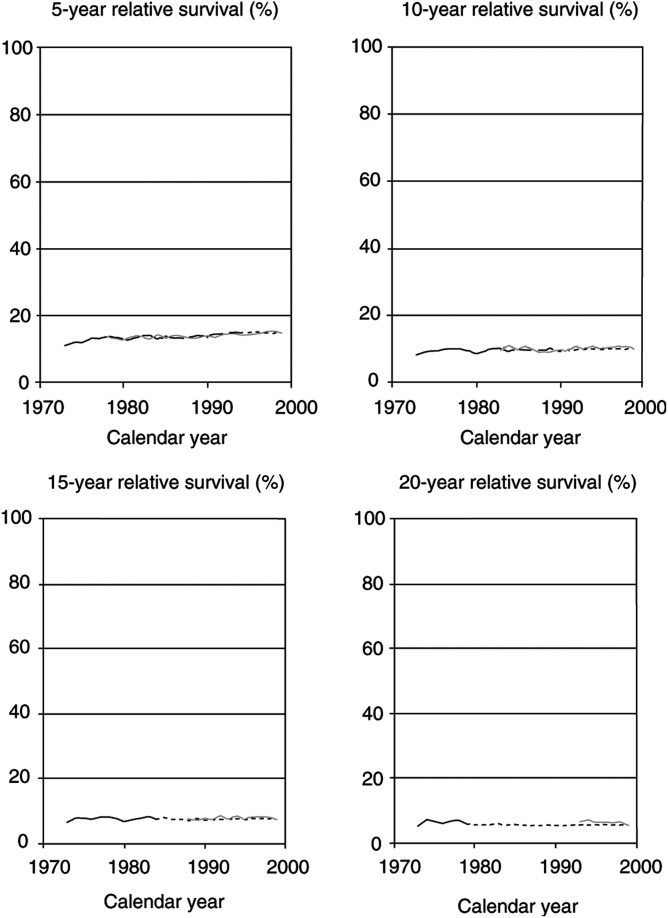
Trends in 5-, 10-, 15-, and 20-year relative survival rates of patients with a first diagnosis of lung cancer in the United States according to cohort analysis (solid black lines), period analysis (solid grey lines), and mixed analysis (dotted extension of black lines). SEER 1973–1999 database, all races, both sexes combined.

, 3

**Figure 3:**
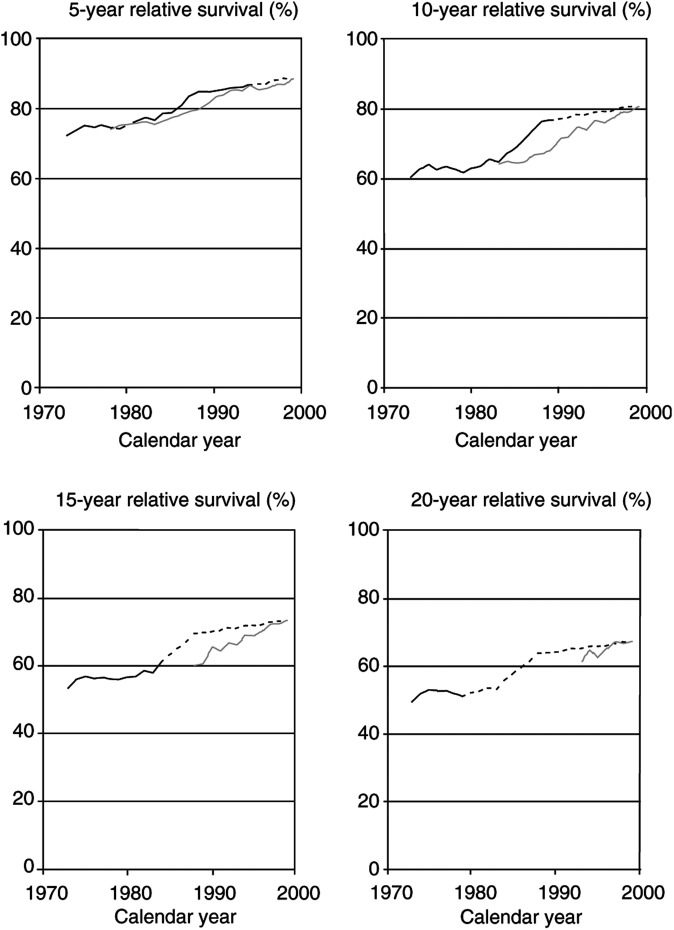
Trends in 5-, 10-, 15-, and 20-year relative survival rates of female patients with a first diagnosis of breast cancer in the United States according to cohort analysis (solid black lines), period analysis (solid grey lines), and mixed analysis (dotted extension of black lines). SEER 1973–1999 database, all races combined.

and 4

**Figure 4:**
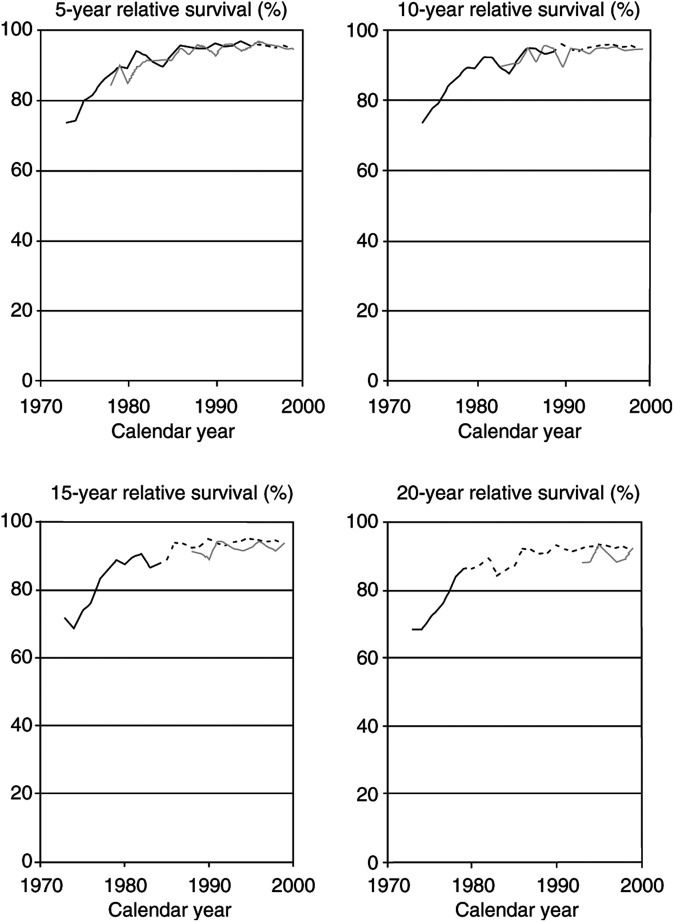
Trends in 5-, 10-, 15-, and 20-year relative survival rates of patients with a first diagnosis of testicular cancer in the United States according to cohort analysis (solid black lines), period analysis (solid grey lines), and mixed analysis (dotted extension of black lines). SEER 1973–1999 database, all races combined.

show analogous analyses of time trends in 5-, 10-, 15-, and 20-year relative survival rates of patients with lung, breast, and testicular cancer.

For lung cancer, prognosis has hardly improved over the past decades, and it continues to be very poor (see Figure 2). As expected from theory (Brenner and Gefeller, 1996, 1997), in this situation cohort and period analyses as well as mixed analysis yield virtually identical estimates for those calendar years for which more than one estimate can be obtained. However, mixed analysis is the only approach that allows for a comprehensive retrospective analysis of the time trends over the past decades.

For breast cancer, a clearly distinct picture emerges (see Figure 3). The overall levels of survival have always been much higher than for both colon and lung cancer, and they further improved over time. On the other hand, the gradient from 5- to 10- to 15- and 20-year relative survival rates is particularly large for this form of cancer, which reflects the relatively high proportion of late cancer deaths among women with breast cancer. Our trend analysis shows that a major improvement in long-term prognosis began with patients diagnosed in the early 1980s. According to the latest cohort estimates as well as the latest period estimates of 5-year survival, the pace of improvement, however, seems to have levelled off in recent years. For patients diagnosed during the years following the onset of rapid improvement, period estimates available then would have somewhat lagged behind the long-term survival rates observed for patients diagnosed in those years many years later, as indicated by their discrepancy from both cohort and mixed estimates. By contrast, the different types of estimates are quite close for the years before the onset and after the levelling off of major improvement.

With respect to testicular cancer, patients diagnosed in the 1980s and 1990s have experienced much higher long-term survival rates than patients diagnosed in the 1970s (see Figure 4). For this form of cancer, 5- and 10-year survival rates around 95% and 15- and 20-year survival rates around 90% have now been achieved. Cohort analysis alone would clearly demonstrate the rapid increase in survival rates of patients diagnosed in the earlier years included in this database, whereas period analysis shows the continuously high levels of survival maintained in the more recent years. Again, the most comprehensive picture of the time trends is provided by mixed analysis.

For the sake of clarity, only point estimates of relative survival are shown in Figures 1,2,3 and 4. With the exception of testicular cancer, the standard errors of these estimates are generally small. Standard errors for all estimates of 5-, 10-, 15-, and 20-year survival are ⩽0.9, ⩽1.1, ⩽1.3, and ⩽1.6% for colon cancer, ⩽0.4, ⩽0.4, ⩽0.5, and ⩽0.5% for lung cancer, ⩽0.7, ⩽0.8, ⩽0.9, and ⩽1.0% for breast cancer, and ⩽3.0, ⩽3.2, ⩽3.5, and ⩽3.8% for testicular cancer, respectively.

## DISCUSSION

This paper illustrates how traditional cohort analysis and the more recently introduced period analysis (Brenner and Gefeller, 1996,^1997^) can be combined to extend retrospective time trend analyses of long-term survival rates. The resulting ‘mixed analysis’ allows a more comprehensive assessment of long-term progress in the prognosis of cancer patients from the earliest to the most recent years of cancer registration.

Retrospective analyses of trends in long-term survival rates over extended time intervals are performed from time to time by many cancer registries with a long history of registration (e.g., Adami *et al*, 1989; Nab *et al*, 1994; Wingo *et al*, 1998; Dickman *et al*, 1999). Typically, such time trend analyses should provide a comprehensive evaluation of time trends encompassing the broadest possible time span from the earliest to the most recent years of cancer registration. This is the context in which ‘mixed analysis’ should be most useful. For other purposes, preferences may be different. For example, ‘pure cohort analysis’ is entirely sufficient, and there is no need of extension, for ‘historical’ assessment of long-term prognosis of cohorts of patients who have been under observation over the full follow-up time of interest. On the other hand, ‘pure period analysis’ might be the preferred method for concurrent monitoring of very recent time trends in long-term survival as well as for deriving the most up-to-date estimates of long-term survival rates at a given time. Period analysis has meanwhile been applied for the latter purpose in different cancer registries (e.g., Brenner *et al*, 1998, 1999, 2001; Brenner and Hakulinen, 2001; Aareleid and Brenner, 2002; Brenner, 2002; Smith *et al*, 2003), whereas mixed analysis has, with very few exceptions (Kaatsch *et al*, 2000; Burkhardt-Hammer *et al*, 2002), not been applied to analyses of time trends in long-term cancer patient survival so far.

Another option that has been employed in traditional survival analysis is to include right-censored observations of patients, who have not been observed over the full follow-up time of interest, in the most recent estimates of long-term survival. For example, with this approach, which has been called ‘complete analysis’ (Brenner and Gefeller, 1997) and which has often been used in analyses of the SEER database (e.g., Wingo *et al*, 1998), the most recent 10-year estimates of survival could have been obtained from all patients diagnosed in 1989 or later years rather than from patients diagnosed in 1989 only in our analysis. However, while frequently used for deriving single recent estimates of long-term survival, complete analyses are usually not used for retrospective assessment of time trends, which have almost exclusively relied on cohort analysis in the past. Furthermore, although complete analysis would have led to a somewhat more up-to-date (and also somewhat more precise) most recent estimate of 10-year survival compared to the one obtained with ‘pure cohort analysis’, it would, unlike period analysis or mixed analysis, not have allowed additional analyses of time trends in 10-year survival rates within the 1989–1999 interval.

Although estimates from period analysis available at a given point of time are more up-to-date than traditional estimates of long-term survival rates available at the same point of time, even the period estimates may tend to be somewhat too low in case of a very rapid increase in survival over time (in such a case, the period estimates may ‘lag somewhat behind’ the most recent developments, which become known later). These patterns have been shown by extensive empirical evaluation in previous work (Brenner and Hakulinen, 2002a,2002b; Brenner, 2003), and they were seen for patients diagnosed with breast cancer in the 1980s in the examples shown in this paper. For example, the major increase in the survival of breast cancer patients in the 1980s would only have been disclosed with substantial delay by period analysis had it been performed then (albeit the delay would have been less severe than with traditional survival analysis conducted at that time). In retrospective analyses, estimates from mixed analysis are always the most up-to-date estimates, as they include as much of the actual survival experience of past cohorts as possible. For more recent years, a large part of the mixed estimate is based on period analysis, and mixed analysis and period analysis are the same for the most recently diagnosed patients.

Like other methods of monitoring survival over time, period analysis and mixed analysis do not by themselves reveal the reasons for the increase of survival rates over time. Such reasons may include advancements in therapy as well as earlier detection (in the latter case increases in long-term survival have to be interpreted with caution, as they may occur even if early detection is ineffective in preventing cancer deaths). Obviously, reasons do vary by cancer site. For example, the increase in survival observed for patients with testicular cancer is likely to be mainly due to a breakthrough in therapy (in particular, the inclusion of cis-platin in chemotherapy schemes) (Bosl and Motzer, 1997), whereas the increase in survival rates of patients with breast cancer might reflect both earlier detection and improved therapy (Hermon and Beral, 1996). More detailed analyses, taking additional factors such as stage at diagnosis, treatment, etc. into account, may help to further differentiate possible reasons. Such analyses could be carried out with period analysis and with mixed analysis in the same way as with traditional cohortwise survival analysis.

Another issue to be considered in time trend analyses of long-term survival rates is age adjustment of survival rates. On average, cancer patients have become older over the past decades in most countries. As prognosis of patients tends to vary with age for most cancers, trends in crude (unadjusted) survival rates may not adequately disclose the true progress in long-term survival rates over time. This particularly applies to time trends over very long time spans within which ‘ageing’ of the cancer populations may be substantial. The issue is of much less concern for relative survival rates that are presented in this paper than for absolute survival rates, as the former vary much less with age than the latter. However, where necessary, age adjustment is as easily carried out in period and mixed analysis as in cohort analysis.

In summary, the combination of traditional cohortwise analysis with period analysis in the form of mixed analysis may be a useful tool for comprehensive retrospective monitoring of time trends in long-term cancer patient survival from the earliest to the most recent years of cancer registration. Recent development of pertinent user-friendly software (Brenner *et al*, 2002a) should facilitate widespread implementation of this new approach by cancer registries.

## References

[bib1] Aareleid T, Brenner H (2002) Trends in cancer patient survival in Estonia before and after the transition from a Soviet republic to an open market economy. Int J Cancer 102: 45–50 12353233 10.1002/ijc.10663

[bib2] Adami H-O, Sparén P, Bergström R (1989) Increasing survival trend after cancer diagnosis in Sweden: 1960–1984. J Natl Cancer Inst 81: 1640–1647 2795692 10.1093/jnci/81.21.1640

[bib3] Berrino F, Capocaccia R, Estève J, Gatta G, Hakulinen T, Micheli A, Sant M, Verdecchia A (eds) (1999) Survival of Cancer Patients in Europe: The EUROCARE-2 Study, IARC Scientific Publications No. 151. Lyon: International Agency for Research on Cancer

[bib4] Bosl GJ, Motzer RJ (1997) Testicular germ-cell cancer. N Engl J Med 337: 242–253 9227931 10.1056/NEJM199707243370406

[bib5] Brenner H (2002) Long-term survival rates of cancer patients achieved by the end of the 20th century: a period analysis. Lancet 360: 1131–1135 12387961 10.1016/S0140-6736(02)11199-8

[bib6] Brenner H (2003) Up-to-date survival curves of children with cancer. Br J Cancer 88: 1693–1697 12771982 10.1038/sj.bjc.6600947 PMC2377141

[bib7] Brenner H, Gefeller O (1996) An alternative method to monitoring cancer patient survival. Cancer 78: 2004–2010 8909323

[bib8] Brenner H, Gefeller O (1997) Deriving more up-to-date estimates of long-term patient survival. J Clin Epidemiol 50: 211–216 9120515 10.1016/s0895-4356(97)00280-1

[bib9] Brenner H, Gefeller O, Hakulinen T (2002a) A computer program for period analysis of survival. Eur J Cancer 38: 690–695 11916552 10.1016/s0959-8049(02)00003-5

[bib10] Brenner H, Hakulinen T (2001) Long-term cancer survival achieved by the end of the 20th century. Most up-to-date estimates from the nationwide Finnish Cancer Registry. Br J Cancer 85: 367–371 11487267 10.1054/bjoc.2001.1905 PMC2364068

[bib11] Brenner H, Hakulinen T (2002a) Advanced detection of time trends in long-term cancer patient survival: experience from 50 years of cancer registration in Finland. Am J Epidemiol 156: 566–577 12226004 10.1093/aje/kwf071

[bib12] Brenner H, Hakulinen T (2002b) Up-to-date long-term survival curves of patients with cancer by period analysis. J Clin Oncol 20: 826–832 11821467 10.1200/JCO.2002.20.3.826

[bib13] Brenner H, Kaatsch P, Burkhardt-Hammer T, Harms DO, Schrappe M, Michaelis J (2001) Long-term survival of children with leukaemia achieved by the end of the 2nd millennium. Cancer 92: 1977–1983 11745273 10.1002/1097-0142(20011001)92:7<1977::aid-cncr1717>3.0.co;2-w

[bib14] Brenner H, Söderman B, Hakulinen T (2002b) Use of period analysis for providing more up-to-date estimates of long-term survival rates: empirical evaluation among 370 000 cancer patients in Finland. Int J Epidemiol 31: 456–462 11980816

[bib15] Brenner H, Stegmaier C, Ziegler H (1998) Recent improvement in survival of breast cancer patients in Saarland, Germany. Br J Cancer 78: 694–697 9744513 10.1038/bjc.1998.562 PMC2063047

[bib16] Brenner H, Stegmaier C, Ziegler H (1999) Trends in survival of patients with ovarian cancer in Saarland, Germany, 1976–1995. J Cancer Res Clin Oncol 125: 109–113 10190318 10.1007/s004320050250 PMC12201893

[bib17] Burkhardt-Hammer T, Spix C, Brenner H, Kaatsch P, Berthold F, Hero B, Michaelis J (2002) Long-term survival of children with neuroblastoma prior to the neuroblastoma screening project in Germany. Med Pediatr Oncol 39: 156–162 12210443 10.1002/mpo.10132

[bib18] Dickman PW, Hakulinen T, Luostarinen T, Pukkala E, Sankila R, Söderman B, Teppo L (1999) Survival of cancer patients in Finland 1955–1994. Acta Oncol 38(Suppl 12): 1–103 10.1080/028418699432996 10225326

[bib19] Ederer F, Axtell LM, Cutler SJ (1961) The relative survival rate: a statistical methodology. Monogr Natl Cancer Inst 6: 101–121 13889176

[bib20] Hakulinen T (1982) Cancer survival corrected for heterogeneity in patient withdrawal. Biometrics 39: 933–942 7168796

[bib21] Hermon C, Beral V (1996) Breast cancer mortality rates are levelling off or beginning to decline in many western countries: analysis of time trends, age-cohort and age-period models of breast cancer mortality in 20 countries. Br J Cancer 73: 955–960 8611414 10.1038/bjc.1996.171 PMC2074271

[bib22] Kaatsch P, Spix C, Michaelis J (2000), Annual Report 1999. German Childhood Cancer Registry, Mainz, Germany

[bib23] Nab HW, Hop WCJ, Crommelin MA, Kluck HM, van der Heijden LH, Coebergh J-WW (1994) Changes in long term prognosis for breast cancer in a Dutch cancer registry. BMJ 309: 83–86 8038671 10.1136/bmj.309.6947.83 PMC2540542

[bib24] Sankaranarayanan R, Black RJ, Parkin DM (eds) (1998) Cancer Survival in Developing Countries, IARC Scientific Publications No. 145. Lyon: International Agency for Research on Cancer 10194635

[bib25] Smith LK, Lambert PC, Jones DR (2003) Up-to-date estimates of long-term cancer survival in England and Wales. Br J Cancer 89: 74–76 12838303 10.1038/sj.bjc.6600976 PMC2394208

[bib26] Surveillance, Epidemiology, and End Results (SEER) Program Public-Use Data (1973–1999) (2002) Bethesda (MD), National Cancer Institute, DCCPS, Cancer Surveillance Research Program, Cancer Statistics Branch, released April 2002, based on the November 2001 submission

[bib27] Wingo PA, Gloeckler Ries LA, Parker SL, Heath CW (1998) Long-term cancer patient survival in the United States. Cancer Epidemiol Biomark Prev 7: 271–282 9568781

